# Neuroimmunological impact of media-induced stress: rethinking inflammation and brain-immune crosstalk

**DOI:** 10.3389/fpsyt.2025.1652541

**Published:** 2025-08-28

**Authors:** Sobi Thomas, K. M. Marykutty, M. Vijayakumar, Anson Thomas, A. R. Gilbert, Carmel Maria Jose

**Affiliations:** ^1^ School of Social Science, Arts and Humanities, Lincoln University College, Petaling Jaya, Malaysia; ^2^ Department of Communication and Media Studies, Marian College Kuttikkanam, Kuttikkanam, India; ^3^ Department of Emergency Medicine, Jaya Matha Hospital Koothattukulam, Ernakulam, India

**Keywords:** media-induced stress, neuroinflammation, HPA axis, brain-immune interaction, microglial priming, cytokines, mental health immunology

## 1 Introduction: Media stress as an emerging neuroimmune challenge

Over the past decade, the pervasive influence of digital media has restructured not just our information environment, but potentially our biological systems. Social media, 24-hour news cycles, and algorithmically amplified crisis content now form a persistent backdrop to modern life. Recent studies show that repeated exposure to emotionally charged news—whether about war, pandemics, or climate disasters—can provoke prolonged psychological stress, which in turn may impact immune function (1). While media psychology has studied the behavioral consequences of such exposure, its immunological ramifications remain underexplored.

This opinion article argues that chronic media-induced psychological stress constitutes a distinct immunological stressor, with measurable effects on neuroinflammatory signaling, blood–brain barrier (BBB) integrity, and central nervous system (CNS) immune surveillance. We propose that this form of stress exposure deserves attention from immunologists not merely as a psychosocial factor, but as a potential driver of low-grade systemic inflammation and neuroimmune dysregulation, especially in vulnerable populations. While this opinion focuses on general neuroimmune impacts, we emphasize the relevance of media-induced stress in the context of multiple sclerosis (MS), a chronic neuroinflammatory disease. Emerging research suggests that stress and immune dysregulation may influence disease onset and flare-ups, making MS a relevant disease model for this discussion (2).

## 2 Brain–immune crosstalk under stress: The biological logic

The brain and immune system operate as a bidirectional network, engaging in a tightly regulated dialogue through neuroendocrine and cytokine pathways. The hypothalamic–pituitary–adrenal (HPA) axis and sympathetic nervous system (SNS) play critical roles in initiating immune responses to psychological stimuli. When the brain perceives a threat—whether physical or symbolic—glucocorticoids and catecholamines are released, modulating cytokine production, lymphocyte trafficking, and microglial activity (3).

However, persistent stress—particularly from unresolved or ambiguous threats as commonly portrayed in media—leads to HPA axis dysregulation (4). Cortisol feedback inhibition becomes impaired, resulting in prolonged elevation of inflammatory mediators, including IL-6, TNF-α, and C-reactive protein (CRP) (5). This biochemical milieu primes the innate immune system while also disrupting adaptive responses, increasing susceptibility to viral infections and autoimmunity.

Importantly, the blood–brain barrier, normally a shield for the CNS, becomes compromised under chronic stress conditions. Animal models show increased permeability and elevated expression of endothelial adhesion molecules following repeated social defeat or immobilization stress. This facilitates peripheral cytokines and immune cells crossing into the CNS, where they interact with microglia, the resident immune sentinels of the brain (6). In autoimmune disorders such as MS, these mechanisms are particularly relevant: stress-induced blood–brain barrier (BBB) permeability and peripheral immune infiltration are hypothesized to trigger demyelinating episodes (7).

## 3 Microglial priming and the stress–inflammation loop

Microglia, the innate immune cells of the CNS, typically remain in a surveillant state, dynamically sampling their environment. Under pathological or stress conditions, they can polarize into M1 (pro-inflammatory) or M2 (anti-inflammatory) states, depending on the context (8). In stress-induced scenarios, however, microglia exhibit a “primed” phenotype—hypersensitive to secondary stimuli and prone to exaggerated cytokine release, even in response to mild challenges.

In rodent models, exposure to chronic unpredictable stress increases Iba1+ microglial density and upregulates expression of TNF-α and IL-1β in hippocampal and prefrontal regions. These alterations correlate with behavioral symptoms analogous to depression and cognitive inflexibility (9). The neuroinflammatory cascade, once triggered, further reinforces HPA axis activation—creating a self-sustaining stress–inflammation loop.

This mechanism has implications for psychiatric and neurodegenerative conditions. Primed microglia have been implicated in neuropsychiatric conditions such as major depressive disorder (MDD) and generalized anxiety disorder (GAD), and they may also play a role in neuroinflammatory conditions like multiple sclerosis (MS), where microglial overactivation contributes to demyelination. (10). In this light, repeated media stress exposure may function as an environmental primer, particularly in individuals with genetic or epigenetic vulnerabilities. This priming process may be particularly harmful in MS, where pro-inflammatory microglial activation is associated with demyelination and neurodegeneration. Stress-induced microglial shifts could potentially exacerbate inflammatory responses in individuals predisposed to or living with MS (11).

## 4 Media-induced stress as an immunological risk factor

The idea that psychological stress can “get under the skin” is not new. However, with the rise of pervasive media, we now face a stressor that is ubiquitous, persistent, and individually targeted. Unlike acute traumas, media-induced stress is chronic, symbolic, and often vicarious—which paradoxically amplifies uncertainty and emotional engagement (12). This creates conditions conducive to low-grade systemic inflammation.

**Figure 1:**
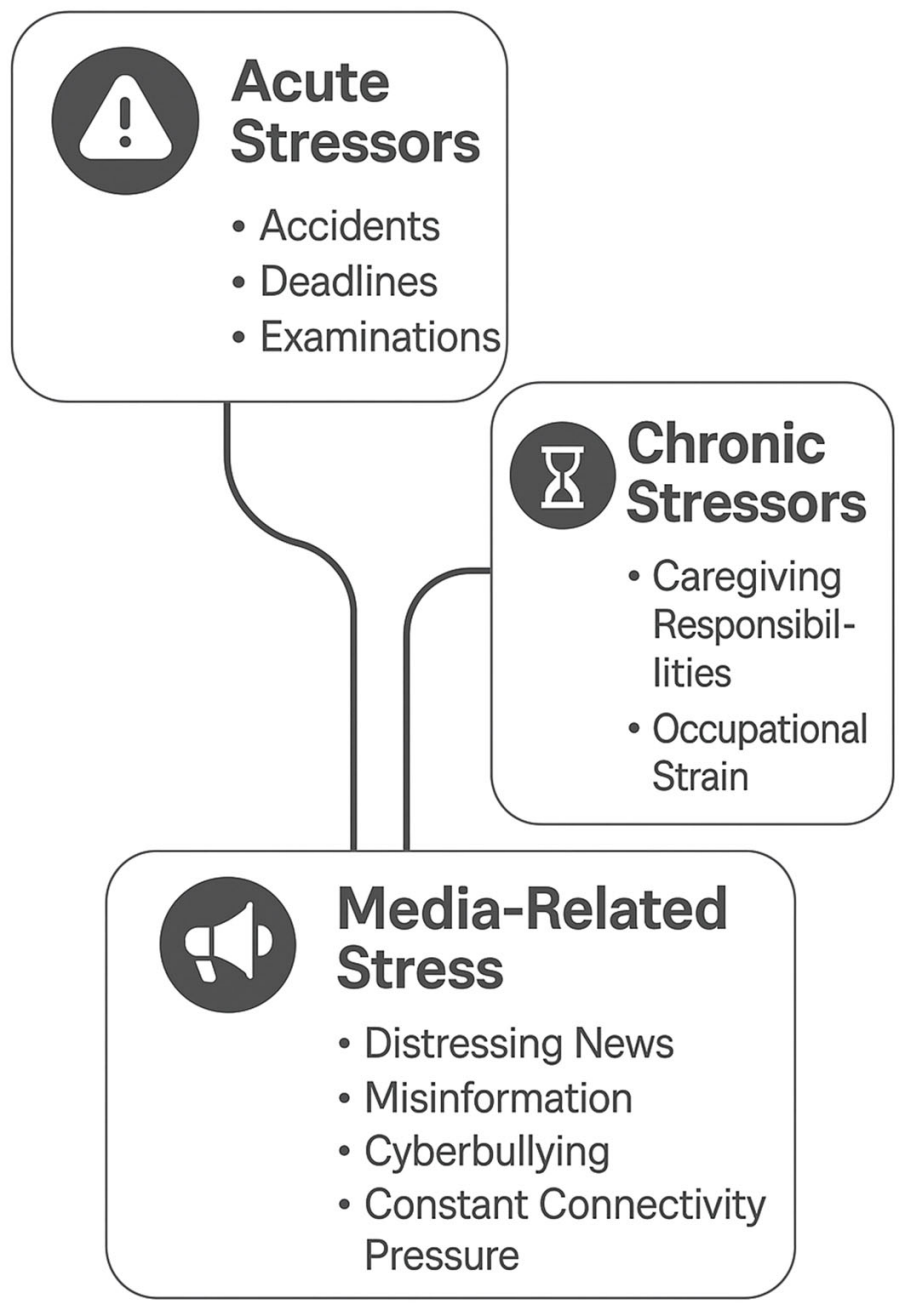
Conceptual model showing acute, chronic, and media-related stressors, with examples and overlaps indicating potential interactions and cumulative effects on neuroimmune function.

For conceptual clarity, stressors can be broadly classified into acute stressors—short-term, discrete events such as accidents, natural disasters, or examinations—and chronic stressors—long-term, ongoing pressures such as caregiving burden, job insecurity, or financial hardship. While acute stressors often provoke a rapid but transient neuroendocrine and immune response, chronic stressors tend to produce prolonged HPA axis activation and low-grade inflammation. Media-related stress can operate in both domains: sudden distressing news or online harassment may act as acute triggers, whereas continuous exposure to algorithmically reinforced negative content may function as a chronic background stressor (13). The three major stressor categories—acute, chronic, and media-related—are depicted in **Figure 1**, showing both unique and overlapping pathways relevant to neuroimmune dysregulation.

Media-induced stress shares features with other chronic symbolic stressors, such as job insecurity, social exclusion, or caregiving burden. These ambiguous, persistent stressors do not involve physical danger, but elicit long-term physiological arousal and immune activation. Including media within this stress category helps contextualize its unique intensity and reach (14). Media-induced stress can be defined as the chronic psychological and physiological arousal resulting from repeated exposure to emotionally charged or threatening digital content— often algorithmically reinforced across digital platforms. Key dimensions include exposure frequency, emotional valence, content type (e.g., violence, health threat), and perceived helplessness (15). This study measured digital stress through self-reported media exposure and emotional response scales, which were correlated with immune assays. It highlights that digital environments may biologically affect immune competence, particularly in young populations.

Recent population studies have linked high media consumption during COVID-19 to elevated IL-6 and CRP levels, poor sleep, and increased autoimmune flares. Digital stress scores correlate with peripheral monocyte activation and impaired Natural Killer (NK) cell cytotoxicity in adolescents and young adults (16). These findings underscore that media exposure is not a neutral behavioral choice, but a potential immunomodulator. In this study, digital stress was assessed via self-reported media exposure, perceived emotional distress, and algorithmic content tracking, which were correlated with blood markers for immune function. Monocyte activity and NK cell assays were performed using peripheral blood samples.

Moreover, emerging data suggest that algorithmically tailored content may worsen immune dysregulation by reinforcing threat perception. Platforms that continuously deliver personalized health fears, geopolitical instability, or climate anxiety contribute to a tonic arousal state, mimicking chronic threat vigilance observed in PTSD (17). This ‘neuroimmune hyperarousal’ may contribute to flare-ups in chronic inflammatory conditions—including neuroimmune disorders such as MS— especially in individuals who are predisposed to or already living with MS.

**Table 1** provides a summary of representative animal and human studies supporting the link between psychological stressors—including media exposure—and immune dysregulation.

**Table 1:** Summary of key human and animal studies linking stress and immune dysregulation.

Study (Citation)	Population/Sample size	Study design	Type of stressor	Biomarkers assessed	Key findings
Bekhbat et al., 2023 (1)	Mice (n not specified)	Experimental	Repeated social defeat	Metabolic markers in splenic immune cells	Chronic stress led to metabolic shifts in innate immunity
Kim et al., 2022 (5)	Diabetic mice (n not specified)	Experimental	HPA axis dysregulation post-stroke	IL-6, TNF-α, CRP	HPA axis dysfunction increased inflammation, worsened stroke outcomes
Martín-Hernández et al., 2023 (6)	Rats (n not specified)	Experimental	Periodontal disease + chronic stress	BBB permeability, endothelial markers	Stress enhanced BBB permeability and CNS immune infiltration
Sales et al., 2024 (9)	Mice (n not specified)	Experimental	Chronic unpredictable mild stress (CUMS)	Iba1+ microglia, IL-1β, TNF-α	CUMS increased microglial priming and depressive behaviors
Naiditch et al., 2025 (16)	Adolescents & young adults (n ≈ 350)	Observational, cross-sectional	High media exposure (COVID-19 period)	Digital stress index, monocyte activation, NK cell cytotoxicity	High media stress correlated with increased inflammation and decreased immune defense
Lindsay et al., 2022 (18)	Older adults (n = 190)	Randomized Controlled Trial (RCT)	Mindfulness intervention (vs. control)	IL-6 production, T-reg activity	MBSR reduced IL-6 and enhanced regulatory immune function
Flintoff et al., 2025 (19)	Human participants (n = 150 planned)	Protocol for longitudinal observational study	Real-world cognitive/physical stress + digital behavior	Wearable HRV, salivary cortisol, IL-6, digital data	Proposed biomarker system for real-time stress assessment
Horikawa et al., 2024 (17)	Mice (n not specified)	Experimental	Chronic restraint stress	Lipid mediators, gene expression in bone marrow/spleen	Stress altered immune gene profiles and lipid mediators

## 5 Toward a framework for biomarker-guided monitoring

Given the accumulating evidence, it is imperative to develop a translational framework to identify and monitor the immunological footprint of media-induced stress. We propose three promising biomarker domains:

1. Circulating cytokines: Longitudinal monitoring of IL-6, IL-1β, TNF-α, and CRP levels can reveal chronic inflammatory tone.
2. Neuroimaging markers: fMRI and Positron Emission Tomography (PET) imaging of microglial activation (e.g., via translocator protein – TSPO - tracers) can track neuroinflammation *in vivo*.
3. Neuroendocrine metrics: Cortisol awakening response and diurnal slope can indicate HPA axis dysregulation.

These measures could be combined into a composite “neuroimmune stress index,” capable of stratifying individuals based on physiological reactivity to media stress. Integrating wearable stress sensors and passive digital behavior data (e.g., doomscrolling patterns, screen time analytics) with immune biomarkers may further refine risk prediction models (19). While the concept of a “neuroimmune stress index” is promising, its widespread implementation is limited by cost and invasiveness. In non-clinical settings, scalable alternatives may include salivary cortisol tracking, digital behavior monitoring, and validated stress questionnaires. Future studies should pilot such models in populations with chronic conditions like MS, where immune modulation is clinically relevant (20).

## 6 Future directions: Toward preventive neuroimmunology

To mitigate the neuroimmunological burden of media-induced stress, several translational pathways can be explored:

- Cognitive immunomodulation: Behavioral interventions such as mindfulness-based stress reduction (MBSR) and cognitive behavioral therapy (CBT) have shown promise in lowering IL-6 and improving regulatory T cell function in stressed individuals (18). Incorporating media hygiene into these protocols—e.g., setting exposure limits or applying algorithmic filters—could optimize outcomes. Examples of media hygiene practices include setting daily screen time limits, muting high-intensity news channels during vulnerable periods, disabling autoplay features, and using content filters to reduce algorithmic amplification of distressing content (21).
- Anti-inflammatory psychopharmacology: Trials using low-dose anti-inflammatory agents (e.g., NSAIDs, minocycline, NLRP3 inhibitors) in depression offer a precedent for targeting inflammation in stress-linked psychiatric disorders. These could be repurposed in high-risk, media-exposed populations pending biomarker validation.
- Precision public health messaging: Immunologists and public health professionals must collaborate with media platforms to develop immune-informed communication strategies—ones that convey risk without triggering excessive alarm, and that reinforce resilience over fear (22).
- AI-assisted immune monitoring: Machine learning models trained on multimodal data (wearable stress signals, social media usage, cytokine trends) can help flag early signs of neuroimmune dysregulation, enabling timely intervention (23).

In addition, physical activity has been shown to exert anti-inflammatory effects and modulate HPA axis function. As a low-cost, accessible intervention, it may buffer against neuroimmune consequences of chronic stress. Integrating media hygiene and physical activity into personalized health strategies could provide effective, non-pharmacological modulation of stress-induced immune activation (24).

## 7 Conclusion

The immunological impact of media-induced stress represents a neglected frontier in neuroimmunology. In a world where digital interfaces shape psychological states, and where stress is increasingly symbolic and chronic, it is no longer sufficient to regard media as merely behavioral or societal (25). It must be seen as a biological exposure pathway—one capable of modifying immune trajectories across the lifespan.

This article calls for a rethinking of brain–immune crosstalk in light of 21st-century stressors. By integrating biomarker discovery, imaging technologies, and digital behavior analytics, we can illuminate the molecular consequences of mediated anxiety and develop precision interventions. Just as we have begun to stratify traumatic brain injury or cancer immunotherapy using spatial and temporal immune data, so too must we map the invisible toll of digital stress.

The future of immunology will not be immune to media. It must embrace its influence—with rigor, ethics, and translational clarity. This is especially relevant in neuroimmune conditions such as multiple sclerosis, where systemic inflammation and BBB disruption can have disease-modifying consequences.
